# Anthony Daniels

**DOI:** 10.1192/bjb.2023.55

**Published:** 2023-10

**Authors:** Abdi Sanati

**Figure:**
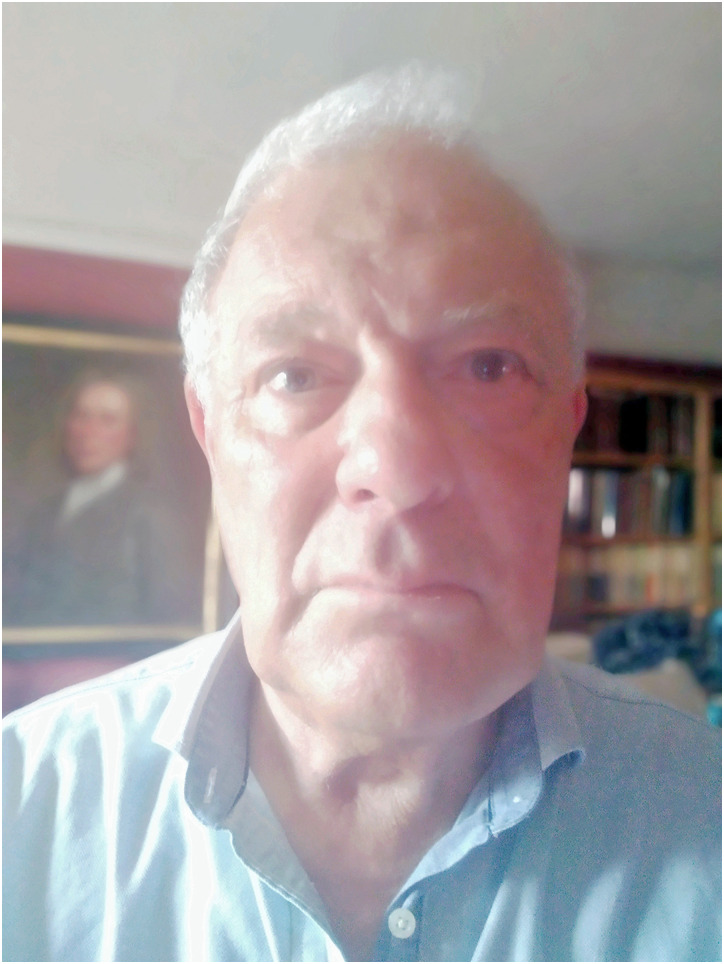

I first met Dr Daniels, who is better known by his pen name Theodore Dalrymple, in a philosophy course, where he delivered a lecture. We engaged in a lively discussion about free will and addiction. Dr Daniels is one of the few true polymaths at present. His writing encompasses a wide range of subjects, including culture, literature, philosophy and travel. He has published several books and articles in reputable journals in and out of medicine. He has even written a book on litter! I admire his clarity of vision and commitment to free enquiry. Dr Daniels is a very interesting author to read. He is even more interesting to disagree with. And that is a very rare quality at the current time.

**Thanks very much Dr Daniels for this interview. You have written about the importance of free will. Do you think free will has been overlooked in psychiatry?**

I think there is always a tension in psychiatry between regarding people as simply the products of things that are beyond their control, for example their genetics and their environment, and people making a choice. And there's always a distinction. I think it's not a good distinction, but there's a distinction between how we regard other people and how we regard ourselves. And while we may regard other people as objects who are just the products of forces around them or in them, we do not think of ourselves in this way. And therefore there's some kind of potential dishonesty in the way we regard people. Having said that, there are circumstances in which people are not responsible for what they do. If you take someone suffering from dementia, behaving in a way that he or she would not have done before, you wouldn't dream of holding that person responsible.

**Free will does have relevance in areas such as addiction and forensics. Do you think that we are ascribing less free will to our patients?**

Yes. I think that certainly in addiction, it is regarded just straightforwardly as a disease, so that you are addicted in the way that you get Parkinson's disease or multiple sclerosis. There's clearly a very big difference, a category difference, between these two types of condition. And I think there is a tendency anyway to reduce or deny that difference. So that, for example, one of the definitions of heroin addiction on the website of the National Institute on Drug Abuse in the United States is that it is a chronic relapsing brain condition. Well, that seems to me a very reduced and reductive way of thinking about addiction.

**I sometimes find this lack of attribution of free will, not holding people as responsible agents, indignifying. I wonder what your opinion is on that.**

I think it dehumanises people to think that they don't have any responsibility. I think that the ultimate cause of this is a decline in religious ways of looking at mankind. Not that I'm really religious myself, I'm not. But we've come to the point where we have these naturalistic explanations and we think that if we attribute free will to people, if we say people have chosen to behave badly, then in effect, what we're saying is we don't want anything to do with them. This seems to me a false dichotomy. If I say that a drug addict is at least partially or largely responsible for his own condition, that is not the same as saying ‘Go away, don't darken my door again. I'm not going to try and help you’. The thing about ascribing responsibility to people is that you see them, in essence, as real human beings, because you don't think of yourself in this way.

**I remember reading the philosopher Hanna Pickard on responsibility without blame. What do you think of that concept?**

I don't think that that's really psychologically possible. If I say that you are responsible for something then I would tend to blame you. Now, there can be mitigating circumstances of course. And any decent system of law or moral philosophy would allow for mitigating circumstances. But you can't mitigate the whole of human behaviour and choice away.

**Recently, we've had a lot of talk on trauma. In psychiatry, I think it has become the overarching narrative and, in a way, people are seen as the products of past traumas. There is no denying the impact that trauma has on people's lives. But my worry is that this narrative will again take agency away.**

I think that that's perfectly possible. And one of the things it potentially does, obviously I don't say it does in all cases, it makes people more vulnerable because they're on the lookout for traumatisation of themselves. This is partly because victims have been sanctified and therefore they actually wish themselves to be victims. And the fact is that, of course, many people are terribly victimised but continue their lives. The idea that for every distress there is an equal and opposite form of therapy or assistance increases the overall level of suffering, at least potentially.

**I go to one of my favourite subjects, risk assessment! What do you think of the preoccupation that we have with risk assessment in psychiatry?**

I dare say that a large part of it comes from attempts to escape responsibility. Once something bad happens, so long as you have done your proper risk assessment, then you can no longer be blamed. Whereas if you haven't done a risk assessment, you can be blamed. Now one of the problems with risk assessments is that they're all statistical and a particularly baleful example of this, in my view, is the granting of parole, which is, of course, completely against the rule of law. It's entirely arbitrary. If you are a prisoner before me, and I say I've done my risk assessment and I think there's an 80% chance of you either committing a crime or not committing a crime, whichever way you look at it, this is completely useless in a law-based system. Obviously I don't think the formalisation of risk assessment has done very much for us. And in fact, in some ways it has made things worse because any protocol in a bureaucratic system becomes an end in itself. People think that once they've filled in some form or other, they've actually done the work. There's nothing further to do. And I've seen this lots of times, endless forms filled in and the most obvious considerations are completely overlooked.

**You mentioned parole, and I know you have written about remorse. Do you think remorse has been overstated?**

I think that the use of remorse in assessing whether a person is going to reoffend is extremely foolish. If you reward people for expressing remorse, then they will express remorse. Whether it has anything to do with their real inner feeling is impossible to say. Actually, what you're doing is rewarding people who are good actors. If people really feel remorse, then this is an internal state, which cannot be encouraged by, for example, giving them a shorter prison sentence.

**Expressing remorse is part of the risk assessments. And I think it does play a role in our dealings with the Ministry of Justice about restricted patients.**

Certainly the more intelligent people will know that they should express remorse. In fact, a friend of mine worked in a prison. Prisoners are not stupid – I mean, many of them are intelligent and cunning. And one of the experienced prisoners said to my friend ‘What you should do if you get a long sentence is to behave very badly to begin with, and gradually you should reduce your bad behaviour, so that the professionals think that you have improved’. I'm not saying that all expressions of remorse are not genuine. But the problem is that it's very difficult to distinguish between a genuine and a fake expression of remorse.

**I want to go back to free will and risk assessment. I remember you have written in one of your papers about metaphysical gaps. I was thinking that we treat risk assessments in much the same way as we predict movement of celestial bodies. As if we can predict the future. And there is a metaphysical gap between the two. Do you think that the concept of free will is causing this gap?**

Whatever you call it, there is a permanent possibility of a human being behaving differently from how you expect or want him to behave. And for whatever reason, I don't think we have ever been able to overcome that. And in effect, I hope we never will be able to, because the idea of total control and understanding of people is, to me, a horrible one.

**I remember Viktor Frankl wrote in *Man's Search for Meaning* that you can understand the mechanism of the human psyche, but human is more than psyche.**

I don't think we will ever fully understand ourselves. And I hope we never fully understand ourselves, because if such understanding were possible, it would be possible for some people and not others. Obviously, it would depend on very high intelligence and high levels of information. And there will be lots of people who would not have that level of understanding, and history would suggest that once people have that degree of power they will abuse it. I don't think it's very likely that we will ever have full understanding but, as I said, I hope we do not.

**We touched on forensics earlier and that takes me to the issue of law. In all the years that I have been doing psychiatry, the most important changes in the field haven't come from research or innovation, but mostly from law. A judge makes a ruling and our whole practice is turned upside down. It all ends up with more bureaucracy. Do you think that law has overreached itself in psychiatry?**

Well, you have to remember that you said you've been in psychiatry for 25 years, more or less. I've been out of psychiatry for 18 of those years. So I'm perhaps not the person to answer this. I dare say there's too much law in psychiatry and there might be too much psychiatry in law. Often there's not a good appreciation that the two things are very different. The two fields are very different and have different criteria.

**I also read in one of your articles that doctors are becoming more like operatives of technology. Can you tell me more?**

Certainly. I am not sure about psychiatry. I think psychiatry probably has a bit of technology envy, in the sense that it's still not very technological by comparison with most of medicine. But to give you a very short answer, a physician in the hospital in which I worked said our patients nowadays arrive in hospital and they go straight into a scan, and nobody really takes much notice of the patient in any other way. I suppose this is inevitable to a degree. This tendency is much greater in fields other than psychiatry.

**I was wondering, what future do you see for psychiatry?**

I think there will always be a need for psychiatry.

**That's reassuring.**

Yes, assuming that we don't find the cause of schizophrenia or the straightforward cure of it or anything like that. But I am somewhat disillusioned about psychiatry. This is because it seems to me to have become less humanistic and more mechanical – not technological, but mechanical. After I retired, I did some medico-legal work, including cases in which psychiatric care had allegedly been to blame for outcomes. It seemed to me that one of the things that had happened was that there had been an awful lot of form filling. Most elementary things were overlooked. Forms were filled in. Nobody took any notice of what the forms actually said. There was no continuity of care. Patients often saw several different supposed care workers and this seemed to me not only stupid, but grossly inhumane. If you imagine yourself in the position of a psychiatric patient, you certainly wouldn't want a procession of different people coming and poking about in your mind, and starting from the beginning every time. I don't know how general this is, but I suspect it is pretty general – but you will be in a better position to say whether this is general or not.

**I think of the way we do defensive practice in psychiatry. While in some specialties they do more tests, we complete more forms here.**

One of the things I've noticed is that if a case ends up in a Coroner's Court, because there's been a fatality, what is looked at is whether the procedure has been followed, whether the forms have been filled in or not. I mean, the important thing about a form is whether it contains any useful information or whether anyone takes any notice of the information that is there. The procedure has become what is important. And I suppose that procedure is to psychiatry what technology is to other branches of medicine.

**What I have seen recently is a rise in doctors becoming more activists and very political. I remember that after the *Roe v. Wade* ruling on abortion in the USA, the editors of one of the medical journals said the United States Supreme Court is the biggest danger to world public health. While I am pro-choice, I find this statement absurd.**

I've noticed a lot of this in all the medical journals, frankly. And what alarms me is not that people say these things, but that there is no alternative point of view ever expressed or published. If there were real debate about these things, then it wouldn't worry me that people expressed political views of that nature. But when there is very little contradiction and no possibility of discussing these things in the public sphere, or in the medical journals, then that is alarming. And it's quite clear to me that ... Well, to take that particular ruling as an example – the ruling was referred to the American Constitution, and it did not make abortion illegal. It had nothing to say about whether abortion should be legal or not. What it said was that abortion is not a right guaranteed by the United States Constitution. I've read the whole of the United States Constitution, including the amendments, and it seems to me that you would have to torture the meaning of the document in order to derive a right to abortion from that document – which is not to say that abortion is either right or wrong. The Constitution has no opinion on that matter. There's nothing in the document that states it and that it's right, or that it's wrong, or that the State should permit it, or that they should forbid it. And that was the ruling. You would never guess this from the commentary.

**I think that it shows again that democratic processes are better than court judgments. But I might be wrong.**

Well, I don't know whether they're better but what I would have wanted was some honest – intellectually honest – discussion about this because you can't say that the United States Constitution guarantees this or that thing. It doesn't guarantee, for example, shelter for everyone. And of course, you can always produce an argument or some kind of rationalisation from that document to result in anything that you think is desirable.

**Talking about having counter-arguments reminds me of the recent situation in Britain regarding the COVID lockdown. There was no room for dissent.**

What is perhaps alarming, to me anyway, in this situation is that the government isn't imposing censorship. Censorship doesn't come from the government. Government is not saying you will not be allowed to publish X, Y or Z. It's that there's a kind of zeitgeist ruled by a group of elite who have no appetite for discussion because they are convinced that they are utterly right. The censorship is not formal, nor is it total. Nevertheless, one sometimes has the impression that one is reading *Pravda*.

**Thank you very much for your time.**

